# Heat induces myogenic transcription factors of myoblast cells via transient receptor potential vanilloid 1 (Trpv1)

**DOI:** 10.1002/2211-5463.12550

**Published:** 2018-12-03

**Authors:** Syotaro Obi, Toshiaki Nakajima, Takaaki Hasegawa, Fumitaka Nakamura, Masashi Sakuma, Shigeru Toyoda, Chuwa Tei, Teruo Inoue

**Affiliations:** ^1^ Research Support Center Dokkyo Medical University Tochigi Japan; ^2^ Department of Cardiovascular Medicine Dokkyo Medical University Tochigi Japan; ^3^ Heart Center Dokkyo Medical University Hospital Tochigi Japan; ^4^ Third Department of Internal Medicine Teikyo University Chiba Medical Center Japan

**Keywords:** heat response signaling, skeletal muscle, translation

## Abstract

Exercise generates heat, blood flow, and metabolic changes, thereby inducing hypertrophy of skeletal muscle cells. However, the mechanism by which heat incudes hypertrophy in response to heat is not well known. Here, we hypothesized that heat would induce differentiation of myoblast cells. We investigated the underlying mechanism by which myoblast cells respond to heat. When mouse myoblast cells were exposed to 42 °C for over 30 min, the phosphorylation level of protein kinase C (PKC) and heat shock factor 1 (Hsf1) increased, and the mRNA and protein expression level of heat shock protein 70 (Hsp70) increased. Inhibitors of transient receptor potential vanilloid 1 (Trpv1), calmodulin, PKC, and Hsf1, and the small interfering RNA‐mediated knockdown of Trpv1 diminished those heat responses. Heat exposure increased the phosphorylation levels of thymoma viral proto‐oncogene 1 (Akt), mammalian target of rapamycin (mTOR), eukaryotic translation initiation factor 4E binding protein 1 (Eif4ebp1), and ribosomal protein S6 kinase, polypeptide 1 (S6K1). The knockdown of Trpv1 decreased these heat‐induced responses. Antagonists of Hsp70 inhibited the phosphorylation level of Akt. Finally, heat increased the protein expression level of skeletal muscle markers such as myocyte enhancer factor 2D, myogenic factor 5, myogenic factor 6, and myogenic differentiation 1. Heat also increased myotube formation. Knockdown of Trpv1 diminished heat‐induced increases of those proteins and myotube formation. These results indicate that heat induces myogenic transcription factors of myoblast cells through the Trpv1, calmodulin, PKC, Hsf1, Hsp70, Akt, mTOR, Eif4ebp1, and S6K1 pathway. Moreover, heat increases myotube formation through Trpv1.

**Abbreviations**
Akt: thymoma viral proto‐oncogene 1
AP‐1: activator protein 1
Creb: cAMP responsive element binding protein 1
Eif4ebp1: eukaryotic translation initiation factor 4E binding protein 1
FoxO: forkhead box O
Hsf‐1: heat shock factor 1
Hsp70: heat shock protein 70
IGF‐1: insulin‐like growth factor 1
IL‐6: interleukin 6
Jak: Janus kinase
Mef2d: myocyte enhancer factor 2D
mTOR: mammalian target of rapamycin
Myf5: myogenic factor 5
Myf6: myogenic factor 6
Myod1: myogenic differentiation 1
NO: nitric oxide
PKC: protein kinase C
RA: all‐*trans*‐retinoic acid
S6K1: ribosomal protein S6 kinase, polypeptide 1
Stat: signal transducers and activators of transcription
Trpv1: transient receptor potential vanilloid 1

Exercise generates heat, blood flow, and metabolic changes in skeletal muscle cells, and they exert influences on the activation of skeletal muscle cells 1. Some groups have reported that a continuous heat exposure of 39 °C increased the mouse myotube diameter 2 and a continuous exposure of 40.5 °C for 48 h increased protein synthesis while decreased protein degradation in porcine muscle satellite cells 3. Heat exposures of 41 or 42 °C for an hour induced the skeletal muscle hypertrophy in rats 4, 5, 6. We have demonstrated that mouse myoblast cells sense heat and produce interleukin 6 (IL‐6) through transient receptor potential vanilloid 1 (Trpv1)/protein kinase C (PKC)/cAMP responsive element binding protein 1 (Creb) signaling pathways 7. When myoblast cells were exposed to temperatures ranging from 37 to 42 °C, the intracellular calcium flux increased temperature‐dependently. Heat also increases the mRNA expression of IL‐6 in a temperature‐dependent manner. IL‐6 is known as an inflammatory cytokine; however, it also plays important roles of anti‐inflammation, immune responses, and metabolic functions 8. IL‐6 activates the Janus kinase (Jak)/signal transducers and activators of transcription (Stat) pathways, activates muscle‐specific transcription factors such as myogenic differentiation 1 (Myod1) and myogenin, and induces differentiation of myoblast cells and fusion of myotubes 9, 10, 11, 12. It suggests that heat induces differentiation of myoblast cells by the paracrine and autocrine signaling, IL‐6/Jak/Stat signaling pathways. In addition to PKC and Creb, heat increases IL‐6 production through c‐Jun NH_2_‐terminal kinase 13, 14, 15, activator protein 1 (AP‐1), and heat shock factor 1 (Hsf‐1) 16. These signals may also induce differentiation of skeletal muscle cells in direct pathways; however, the mechanism in response to heat is not well known.

Sarcopenia is defined as a syndrome characterized by progressive and generalized loss of skeletal muscle mass and strength with a risk of adverse outcomes such as physical disability, poor quality of life, and death 17. Genetic mutant mouse models have been developed to determine the biochemical and physiological pathways which influence the processes of sarcopenia 18. Insulin‐like growth factor 1 (IGF‐1) 19, thymoma viral proto‐oncogene 1 (Akt) 20, and mammalian target of rapamycin (mTOR) 21 increase the protein production. On the other hand, forkhead box O (FoxO) 22, muscle‐specific ring finger protein 1/muscle atrophy f‐box 23, myostatin 24, and apoptosis signaling 25 increase the protein degradation. These molecules influence the balance of protein production and degradation among them. The countermeasures against sarcopenia should be taken urgently in aging societies. The effective method for prevention and treatment is exercise; however, it is difficult for people with sarcopenia to achieve the effective rehabilitation training.

Considering that heat has pleiotropic effect, we speculated that heat induces myogenic transcription factors of myoblast cells as the direct effect. We investigated the signal transduction pathway in response to heat.

## Results

### Heat activates PKC and Hsf1 through Trpv1

**Figure 1:**
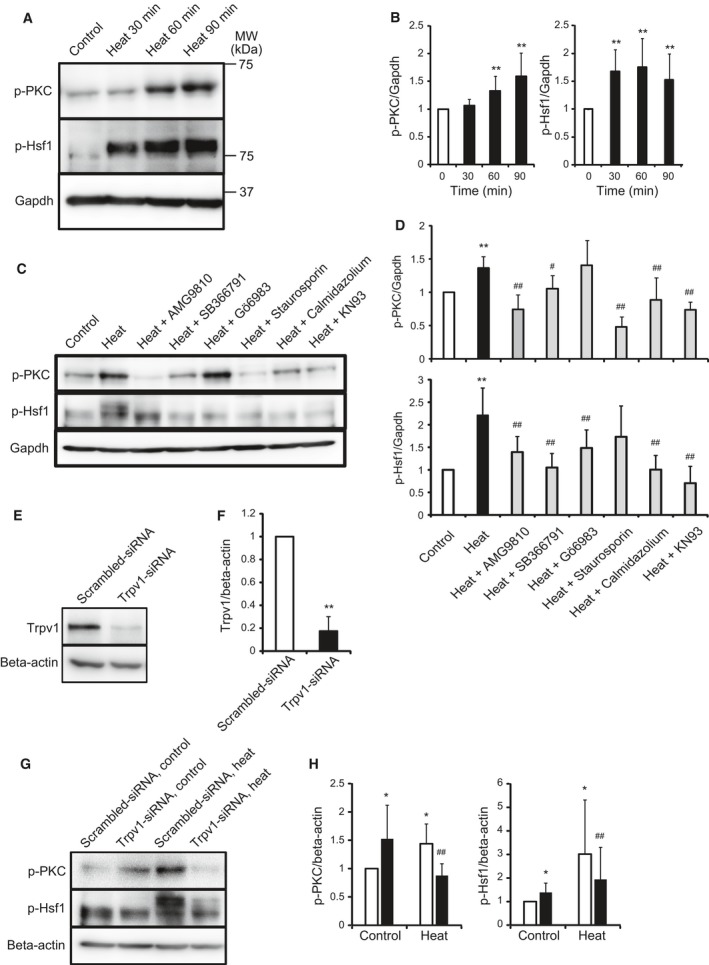
Effect of heat on the Trpv1, PKC, and Hsf1 signaling pathway. The phosphorylation level of PKC and Hsf1 exposed to 42 °C (heat) for various time was analyzed (A). Quantitative analysis of A (B). Values are means ± SD of six independent experiments. **P <* *0.01 vs 37 °C control. Cells were added with 0.1% DMSO and various inhibitors, and were incubated at 42 °C (heat) for 30 min (PKC) or 25 min (Hsf1), and the phosphorylation level of PKC and Hsf1 was analyzed (C). Quantitative analysis of C (D). Values are means ± SD of six independent experiments. **P <* *0.01 vs 37 °C (control), #*P *<* *0.05, ##*P *<* *0.01 vs heat. Cells were transfected with scrambled‐siRNA and Trpv1‐siRNA, and then, the protein expression levels of Trpv1 were analyzed by western blotting (E). The intensity of TRPV1 was analyzed quantitatively (F). Values are means ± SD of five independent experiments. **P <* *0.01 vs scrambled‐siRNA. Cells were transfected with scrambled‐siRNA (white bar) and Trpv1‐siRNA (black bar) and were incubated at 37 °C (control) or 42 °C (heat) for 30 min, and then, the phosphorylation level of PKC and Hsf1 was analyzed (G). Quantitative analysis of E (H). Values are means ± SD of five independent experiments. *P <* *0.05 vs scrambled‐siRNA at 37 °C, ##*P *<* *0.01 vs scrambled‐siRNA at 42 °C.

To investigate the mechanism of heat‐induced PKC signaling, C2C12 mouse myoblast cells were exposed to heat for 30, 60, and 90 min, and the phosphorylation level of PKC, a calcium binding protein, was analyzed by western blotting. Heat applied over 30 min increased the phosphorylation level of PKC (Fig. 1A,B). The Trpv1 antagonists, AMG9810 and SB366791, PKC antagonists, Gö6983 and staurosporine, and calmodulin kinase antagonists, calmidazolium and KN93, were added with the medium of cells, and cells were exposed to the temperature of 42 °C for 30 min. AMG9810, SB366791, staurosporine, calmidazolium, and KN93 but not Gö6983 decreased the heat increased phosphorylation level of PKC. This suggests that PKC is activated by Trpv1 and calmodulin signaling pathways (Fig. 1C,D). The phosphorylation level of Hsf1, a key effector of heat shock protein gene transcription, was analyzed. The temperature of 42 °C for 30, 60, and 90 min increased the phosphorylation level of Hsf1 (Fig. 1A,B). Heat exposure was performed after the addition of AMG9810, SB366791, Gö6983, staurosporine, calmidazolium, and KN93. Those antagonists without staurosporine decreased the phosphorylation level of Hsf1 induced by heat, suggesting that Hsf1 is activated by Trpv1, calmodulin, and PKC signaling pathways (Fig. 1C,D). The Trpv1 gene knockdown assay was performed to clarify whether the heat‐induced phosphorylation of PKC and Hsf1 is dependent on Trpv1. The Trpv1 knockdown efficiency using the Trpv1‐small interfering RNA (siRNA) transfection was analyzed by western blotting. The protein expression level of Trpv1 in cells transfected with the Trpv1‐siRNA was significantly reduced compared with that of cells transfected with the scrambled‐siRNA (Fig. 1E,F). Myoblast cells transfected with the Trpv1‐siRNA and scrambled‐siRNA were exposed to 42 °C for 30 min, and the phosphorylation levels of PKC and Hsf1 were analyzed. The Trpv1 knockdown inhibited the heat‐induced increase of phosphorylation level of PKC and Hsf1 (Fig. 1G,H).

These findings indicate that Trpv1 senses heat and transmits the signal inside myoblast cells through the calcium signaling, calmodulin, PKC, and Hsf1.

### Heat produces Hsp70 via Trpv1

**Figure 2:**
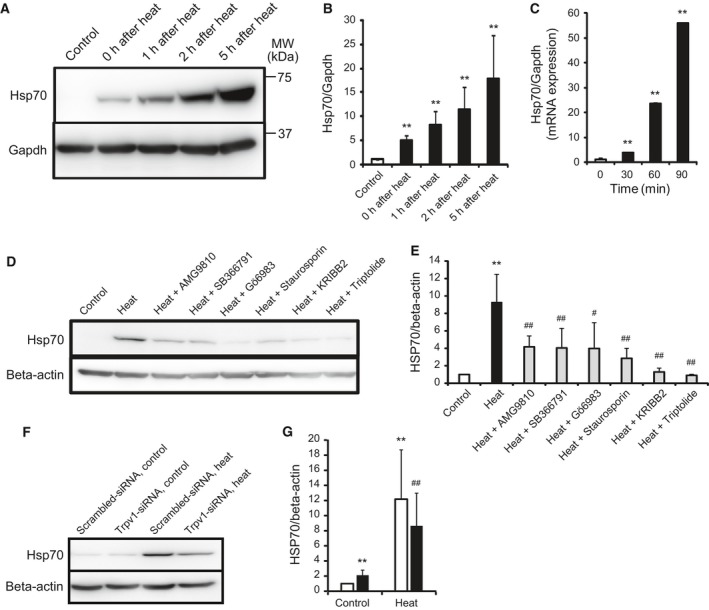
Effect of heat on the Hsp70 production. Cells were exposed to 42 °C for 2 h and were incubated at 37 °C for various time, and then, the protein expression level of Hsp70 was analyzed. Quantitative analysis of A (B). Values are means ± SD of five independent experiments. **P <* *0.01 vs 37 °C (control). The mRNA expression level of Hsp70 after heat exposure of 42 °C for various time was analyzed (C). Values are means ± SD of five independent experiments. **P <* *0.01 vs 37 °C control. Cells were added with 0.1% DMSO and various inhibitors, and were incubated at 42 °C for 2 h following 37 °C for 30 min (heat), and the protein expression level of Hsp70 was analyzed (D). Quantitative analysis of D (E). Values are means ± SD of five independent experiments. **P <* *0.01 vs 37 °C (control), #*P *<* *0.05, ##*P *<* *0.01 vs heat. Cells were transfected with scrambled‐siRNA (white bar) and Trpv1‐siRNA (black bar) and were incubated at 37 °C (control) or 42 °C (heat) for 2 h, and then, the protein expression level of Hsp70 was analyzed (F). Quantitative analysis of F (G). Values are means ± SD of five independent experiments. **P <* *0.01 vs scrambled‐siRNA at 37 °C, ##*P *<* *0.01 vs scrambled‐siRNA at 42 °C.

Cells exposed to the temperature of 42 °C for 2 h were incubated at 37 °C for 1, 2, and 5 h, and the protein expression level of heat shock protein 70 (Hsp70), a target of Hsf1, was analyzed. Heat exposure increased the protein expression level of Hsp70 time‐dependently (Fig. 2A,B). Cells were exposed to the temperature of 42 °C for 30, 60, and 90 min, and the mRNA expression level of Hsp70 was analyzed by real‐time PCR. The mRNA expression level of Hsp70 also increased time‐dependently (Fig. 2C). AMG9810, SB366791, Gö6983, staurosporine, and Hsf1 antagonists, KRIBB2 and triptolide, were added with the medium of cells, and cells were exposed to the temperature of 42 °C for 2 h following the incubation of 37 °C for 30 min. Every antagonist decreased the protein expression level of Hsp70 induced by heat (Fig. 2D,E). Cells transfected with the Trpv1‐siRNA and scrambled‐siRNA were exposed to 42 °C for 2 h, and the protein expression level was analyzed. The Trpv1 knockdown inhibited the heat‐induced increase of Hsp70 (Fig. 2F,G).

These findings indicate that heat produces Hsp70 through Trpv1, calmodulin, PKC, and Hsf1 signaling pathways.

### Heat activates Akt, mTOR, Eif4ebp1, and S6K1 signaling pathways

**Figure 3:**
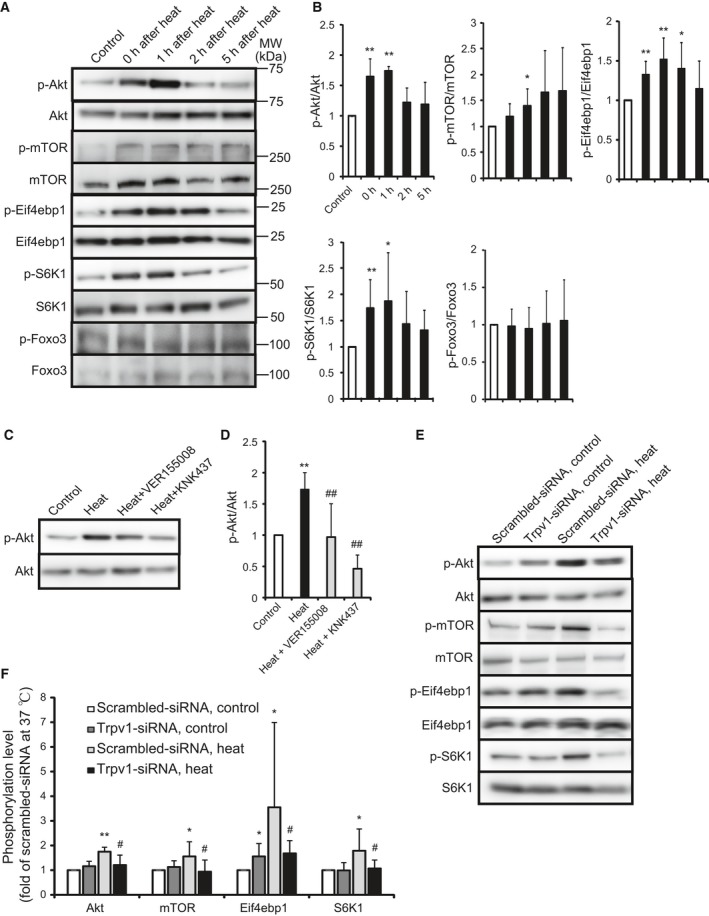
Effect of heat on the Akt, mTOR, Eif4ebp1, and S6K1 signaling pathway. The phosphorylation level of Akt, mTOR, Eif4ebp1, S6K1, and Foxo3 exposed to 42 °C for 2 h (heat) following 37 °C for various time was analyzed (A). Control shows non‐heat‐treated cells. Quantitative analysis of A (B). Values are means ± SD of five independent experiments. *P <* *0.05, **P <* *0.01 vs non heat‐treated cells (control). Cells were added with 0.1% DMSO and various inhibitors and were incubated at 37 °C (control) or 42 °C for 90 min (heat), and the phosphorylation level of Akt was analyzed (C). Quantitative analysis of C (D). Values are means ± SD of six independent experiments. **P <* *0.01 vs 37 °C control, ##*P *<* *0.01 vs heat. Cells were transfected with scrambled‐siRNA (white bar) and Trpv1‐siRNA (black bar) and were incubated at 37 °C (control) or 42 °C (heat) for 60 min (Akt and mTOR) and 90 min (Eif4ebp1 and S6K1), and then, the phosphorylation level was analyzed (E). Quantitative analysis of E (F). Values are means ± SD of five independent experiments. *P <* *0.05, **P <* *0.01 vs scrambled‐siRNA at 37 °C, #*P *<* *0.05 vs scrambled‐siRNA at 42 °C.

Myoblast cells exposed to the temperature of 42 °C for 2 h were incubated at 37 °C for 1, 2, and 5 h, and the phosphorylation levels of Akt, mTOR, eukaryotic translation initiation factor 4E binding protein 1 (Eif4ebp1), ribosomal protein S6 kinase, polypeptide 1 (S6K1), and Foxo3, which are related to degradation, were analyzed. Heat exposure increased the phosphorylation levels of Akt, mTOR, Eif4ebp1, and S6K1, while it did not change that of Foxo3 (Fig. 3A,B). Cells added with HSP70 antagonists, VER155008 and KNK437, were exposed to the temperature of 42 °C for 90 min, and the phosphorylation level of Akt was analyzed. Every antagonist decreased the heat‐induced increase of Akt phosphorylation (Fig. 3C,D). Cells transfected with the Trpv1‐siRNA and scrambled‐siRNA were exposed to heat, and the phosphorylation levels of Akt, mTOR, Eif4ebp1, and S6K1 were analyzed. The Trpv1 knockdown diminished the heat response (Fig. 3E,F).

### Heat induces the skeletal muscle markers

**Figure 4:**
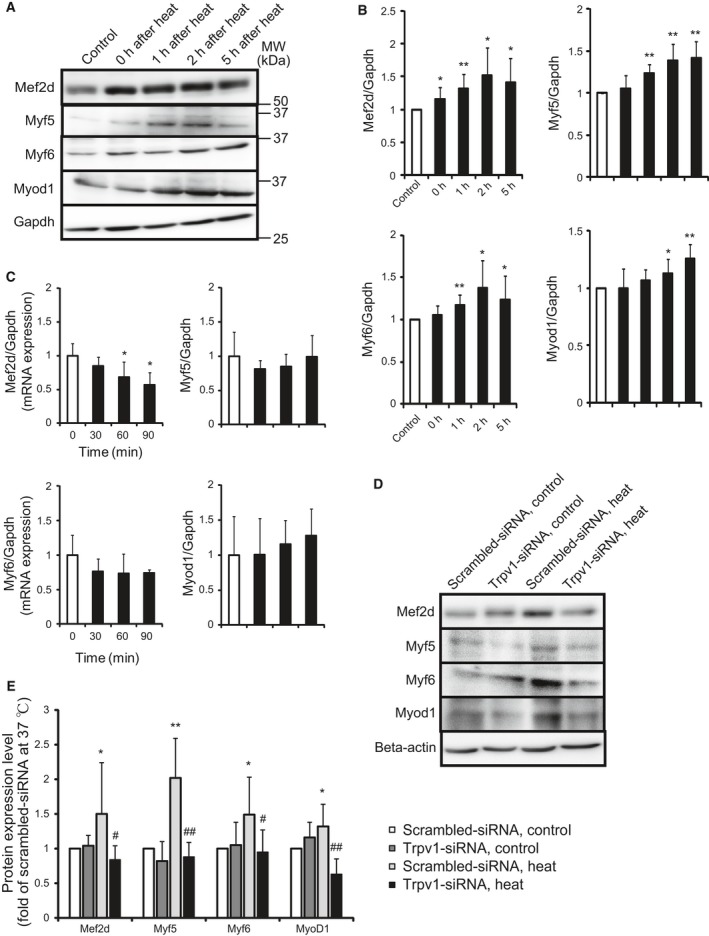
Effect of heat on the differentiation of myoblast cells. Cells were exposed to 42 °C for 2 h and were incubated at 37 °C for various time, and then, the protein expression level of Mef2d, Myf5, Myf6, and Myod1 was analyzed (A). Control shows non‐heat‐treated cells. Quantitative analysis of A (B). Values are means ± SD of five independent experiments. *P <* *0.05, **P <* *0.01 vs non‐heat‐treated cells (control). The mRNA expression level after heat exposure of 42 °C for various time was analyzed (C). Values are means ± SD of five independent experiments. *P <* *0.05 vs 37 °C. Cells were transfected with scrambled‐siRNA (white bar) and Trpv1‐siRNA (black bar) and were incubated at 37 °C (control) or 42 °C for 2 h following 1 h (Myf6) and 2 h (Mef2d, Myf5 and Myod1; heat), and then, the protein expression level was analyzed (D). Quantitative analysis of D (E). Values are means ± SD of five independent experiments. *P <* *0.05, **P <* *0.01 vs scrambled‐siRNA at 37 °C, #*P *<* *0.05, ##*P *<* *0.01 vs scrambled‐siRNA at 42 °C.

Myoblast cells exposed to the temperature of 42 °C for 2 h were incubated at 37 °C for 1, 2, and 5 h, and the protein expression levels of myocyte enhancer factor 2D (Mef2d), myogenic factors 5, 6 (Myf5,6), and MyoD1, transcription factors related to the differentiation of myoblast cells, were analyzed. Heat increased every protein expression level (Fig. 4A,B). Next, myoblast cells were exposed to the temperature of 42 °C for 30, 60, and 90 min, and the mRNA expression levels of Mef2d, Mef5, Mef6, and MyoD1 were analyzed. Heat decreased the mRNA expression level of Mef2d and did not change that of Myf5, Myf6, and MyoD1 (Fig. 4C). Myoblast cells transfected with the Trpv1‐siRNA and scrambled‐siRNA were exposed to heat, and the protein expression levels of Mef2d, Myf5, Myf6, and Myod1 were analyzed. The Trpv1 knockdown inhibited the heat‐induced increase of every marker (Fig. 4D,E). This indicates that heat induces Mef2d, Myf5, Myf6, and Myod1 by increasing the translation level through Trpv1.

### Heat induces myotube formation

**Figure 5:**
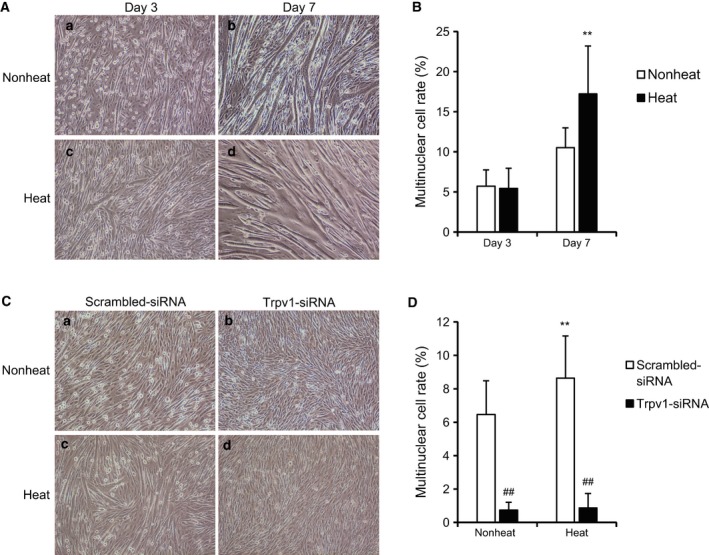
Effect of heat on the myotube formation. Cells were exposed to 42 °C for an hour (heat), were incubated at 37 °C in differentiation medium for 3 and 7 days, and then were observed by a microscope (A, ×10, –a: nonheat at day 3, –b: nonheat at day 7, –c: heat at day 3, and –d: heat at day 7). Fusion index was analyzed (B). Values are means ± SD of five independent experiments. **P <* *0.01 vs non‐heat‐treated cells. Cells were transfected with scrambled‐siRNA (white bar) and Trpv1‐siRNA (black bar), were incubated at 37 °C (nonheat) or 42 °C for an hour (heat) following 3 days, and were observed by a microscope (C, ×10, –a: nonheat with scrambled‐siRNA, –b: nonheat with Trpv1‐siRNA, –c: heat with scrambled‐siRNA, and –d: heat with Trpv1‐siRNA). Fusion index was analyzed (D). Values are means ± SD of five independent experiments. **P <* *0.01 vs scrambled‐siRNA at nonheat, ##*P *<* *0.01 vs scrambled‐siRNA at heat.

To investigate the potential role of heat in myoblast differentiation, myoblast cells were exposed to the temperature of 42 °C for an hour and their differentiation status was assessed by measuring the fusion index. Myotubes were formed at 3 days of differentiation and more myoblast cells exposed to heat formed myotubes at 7 days of differentiation compared with nonheat cells (Fig. 5A,B). Next, myoblast cells transfected with the Trpv1‐siRNA and scrambled‐siRNA were exposed to the temperature of 42 °C for an hour, and fusion index was analyzed. The Trpv1 knockdown decreased the basal potential of myotube formation and diminished the heat‐induced increase of myotube formation (Fig. 5C,D). This indicates that heat induces myoblast differentiation by increasing the myotube formation through Trpv1.

## Discussion

**Figure 6:**
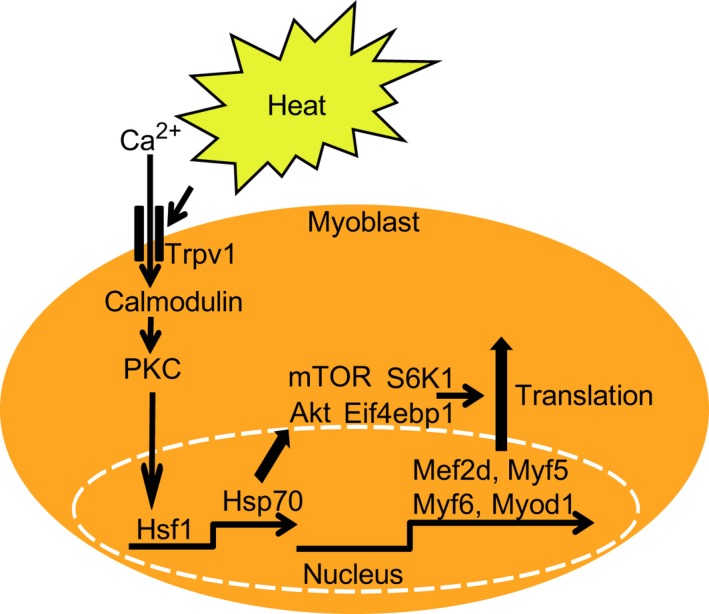
A schematic diagram which heat induces myogenic transcription factors of myoblast cells. Heat activates calmodulin, PKC, Hsf1, Hsp70, Akt, mTOR, Eif4ebp1, and S6K1, and it increases protein expression levels of Mef2d, Myf5, Myf6, and Myod1.

In this study, we demonstrated that heat increases muscle‐specific transcription factors, Mef2d, Myf5, Myf6, and Myod1, in myoblast cells. The mechanism depends on the activation of Trpv1, calmodulin, PKC, Hsf1, Hsp70, Akt, mTOR, S6K1, and Eif4ebp1. Furthermore, heat increases the myotube formation through Trpv1 (Fig. 6).

Trpv1 is a nonselective cation channel which senses circumstance changes such as heat and acidic condition 26, 27. The temperature over 42 °C opens the gate of Trpv1 allowing calcium to flow into cells in nerve cells. Our previous study showed that the temperature over 39 °C increased the intracellular calcium flow in myoblast cells temperature‐dependently 7. In this study, Trpv1 altered the cell signaling events that increase myogenic transcription factors in myoblast cells in response to heat. Trpv1 plays important roles in the process of differentiation. All‐*trans*‐retinoic acid (RA) induces to differentiate Trpv1‐expressing neuroblastoma cells into a neuronal phenotype. RA up‐regulates the protein expression level of Trpv1 in the cell bodies and the developed neuritis 28. Neural progenitor cells derived from Trpv1 knockout mice decreased the mRNA expression level of astrocytic markers glial fibrillary acidic protein and S100 calcium binding protein B, and a neuronal marker beta‐3‐tubulin compared with that of wild mice 29. The mRNA expression level of Trpv1 increases during the cardiac differentiation from embryonic stem cells. The antagonists or knockdown of Trpv1 decreased the calcium influx level, the percentage of spontaneous beating embryonic cells, and cardiomyocyte gene markers including cardiac actin, cardiac troponin T/I, and α myosin heavy chain 30. A Trpv1 antagonist capsazepine inhibits the osteoclastic bone resorption, protects against the ovariectomy induced bone loss in mice, and inhibits the osteoblast activity and bone formation 31, whereas Trpv1 agonist capsaicin inhibits the differentiation of monocyte‐derived dendritic cells 32. Capsaicin increases calcium influx and prevents adipogenesis and down‐regulation of Trpv1 in 3T3‐L1 pre‐adipocytes 33.

Our study revealed that the heat exposure of 42 °C induces Hsp70 through the Trpv1, calmodulin, PKC, and Hsf1 signaling pathways, and induces the expression of myogenic transcription factors. Here, we did not show the blotting for the total expression levels of PKC and Hsf1 to examine the induction of those phosphorylation. The limitations imposed by the differences of internal controls should be borne in mind when evaluating the results. Previous study showed that heat shock proteins contribute to skeletal muscle remodeling. Male Wistar rats of 10 weeks old were assigned to control housed at the temperature of 24 ± 1 °C, eccentric exercise of downhill treadmill running, or heat shock of 41 °C for 20 min 48 h before eccentric exercise. Both the total protein amount and the protein expression level of neonatal myosin heavy chain and Hsp72 in soleus muscles increased in the heat shock group, but did not change in the control or eccentric exercise only groups 34. The weight of soleus muscle and the protein expression level of Hsp72 and Hsp110 in wild‐type mice increased 7 days after the exposure of the temperature of 41 °C for 1 h. On the other hand, those of Hsf1‐null mice did not change after the same heat exposure. Moreover, the absence of Hsf1 gene suppressed heat stress‐associated phosphorylation of Akt and S6K1 in soleus muscles 35. These findings indicate that heat shock proteins play an important role in the remodeling of skeletal muscle cells.

Thymoma viral proto‐oncogene 1, mTOR, Eif4ebp1, and S6K1 signals induce initiation of translation and induce skeletal hypertrophy 36, 37. The Akt phosphorylation is mediated by growth factors such as IGF‐1 and insulin 38, mechanical stress, stretch 39, 40, 41, and heat shock proteins 42. Heat exposure of 41 °C for 30 min increased the phosphorylation level of Akt in rat skeletal muscle 43. MTOR forms the catalytic center of the two signaling complexes, mTORC1 and mTORC2. MTORC1 regulates skeletal muscle protein synthesis 44. The temperature of 41 °C for 48 h increased protein expression level of HSP70 and the phosphorylation level of Akt, mTOR, and S6K in skeletal muscle satellite cells 45. Heat exposure of 41 °C for 30 or 60 min increased the phosphorylation level of S6K in rat skeletal muscle 43, 46, while heat exposure of 44 °C for 20 min inhibited protein synthesis and decreased the phosphorylation levels of Eif4ebp1 in Chinese hamster ovary cells 47. We showed that heat exposure of 42 °C increases the phosphorylation level of Akt, mTOR, Eif4ebp1, and S6K1 in myoblast cells.

Recently, Waon therapy, a thermal therapy in a heated chamber like dry sauna, has been developed and applied for patients with heart failure 48. Possible mechanisms of its effectiveness for heart failure are considered as follows: preload and afterload reduction through peripheral vasodilation, automatic nervous system, oxidative stress reduction, and improvement of endothelial function through increase in endothelial nitric oxide (NO) synthase and NO production. In patients with heart failure, especially in elderly patients, sarcopenia of skeletal muscles is a risk factor for patient health outcomes. From our results, we can envision that hyperthermia such as Waon therapy in combination with rehabilitation training would be a promising treatment for patients with sarcopenia.

In conclusion, heat increases myogenic transcription factors of myoblast cells through the Trpv1, calmodulin, PKC, Hsf1, Hsp70, Akt, mTOR, Eif4ebp1, and S6K1 signaling pathways. Moreover, heat increases the myotube formation through Trpv1.

## Materials and methods

### Materials

The antagonists used were as follows: 10 μm AMG9810 (Alomone, Jerusalem, Israel): a Trpv1 antagonist, 10 μm SB366791 (AdipoGen, San Diego, CA, USA): a Trpv1 antagonist, 1 μm Gö6983 (Cayman Chemical, Ann Arbor, MI, USA): a PKC antagonist, 100 nm staurosporine (LKT, St. Paul, MN, USA): a PKC antagonist, 5 μm calmidazolium (Cayman Chemical): a calmodulin antagonist, 10 μm KN93 (Cayman Chemical): a calmodulin kinase II antagonist, 3 μm KRIBB2 (Tocris Biosciences, Bristol, UK): a Hsf‐1 antagonist, 100 nm triptolide (Cayman Chemical): a Hsf‐1 antagonist, 50 μm VER155008 (Tocris Biosciences): a HSP70 antagonist, and 100 μm KNK437 (Tocris Biosciences): a HSP70 antagonist. All reagents were added into cells with a final concentration of 0.1% DMSO.

### Culture of myoblast

C2C12 mouse myoblast cells were purchased from RIKEN BRC Cell Bank (Tsukuba, Japan). They were cultured at 37 °C under 5% CO_2_ atmosphere in Dulbecco's modified Eagle's medium (DMEM; Nacalai, Kyoto, Japan) with 10% FBS (Gibco, Tokyo, Japan) and 1% Penicillin/Streptomycin/Amphotericin B (Nacalai) on the collagen type I (Nitta Gelatin, Tokyo, Japan) coated dishes for 2 or 3 days. They were passaged 15 times. Differentiation was induced by replacing the growth medium with differentiation medium consisting of DMEM, 2% horse serum (Gibco), and Penicillin/Streptomycin/Amphotericin B (Nacalai). A second incubator was preset to an environment temperature of 42 °C. After dishes were placed in the preset incubator for 2 h, they were replaced in the incubator set at 37 °C. The medium was not changed before and after the heat exposure.

### Myotube analysis

Fusion index was determined by counting the number of multinucleated cell and expressed as a percentage of the total cells including mononucleated cells and multinucleated cells. A microscope (Nikon, Tokyo, Japan, Eclipse TE2000‐U) was used for the observation.

### Transfection of synthetic small interfering RNA (siRNA)

Transient receptor potential vanilloid 1‐siRNA and scrambled siRNA were purchased from Invitrogen (Tokyo, Japan). The Trpv1‐siRNA was transfected into C2C12 cells on the six‐well plate coated with the collagen type I to a final concentration of 16 nm with 0.3% Lipofectamine RNAiMax (Invitrogen) as previously described 7. Transfected cells were incubated for 48 h before being used for experiments.

### Real‐time PCR analysis

Total RNA samples were prepared from cells with GenElute Mammalian total RNA miniprep kit (Sigma Aldrich, Tokyo, Japan), and first‐strand cDNA was generated using a ReverTra Ace qPCR RT Master Mix (Toyobo, Osaka, Japan). After reverse transcription of the RNA into cDNA, real‐time PCR was used to monitor gene expression with a 7300 Real Time PCR System (Applied Biosystems, Tokyo, Japan) according to standard procedures as previously described 49, 50. PCR was performed with SYBR Green Realtime PCR Master Mix‐Plus (Toyobo) and primer pairs for Hsp70, Mef2d, Myf5, Myf6, Myod1, and glyceraldehyde‐3‐phosphate dehydrogenase (Gapdh), respectively. The forward and reverse primer sequences for Hsp70 were 5′‐CCGCCTACTTCAACGACTC‐3′ and 5′‐ TCTTGAACTCCTCCACGAAG‐3′. The forward and reverse primer sequences for Mef2d were 5′‐CATTCATGAGCACAGTGTGA‐3′ and 5′‐GCTCGTTGTACTCGGTGTAC‐3′. The forward and reverse primer sequences for Myf5 were 5′‐CCTGTCTGGTCCCGAAAGAAC‐3′ and 5′‐GACGTGATCCGATCCACAATG‐3′. The forward and reverse primer sequences for Myf6 were 5′‐GATTCTGCGGAGTGCCATCA‐3′ and 5′‐TGTTCCAAATGCTGGCTGAG‐3′. The forward and reverse primer sequences for Myod1 were 5′‐ACATAGACTTGACAGGCCCCGA‐3′ and 5′‐AGACCTTCGATGTAGCGGATGG‐3′. The forward and reverse primer sequences for Gapdh were 5′‐TGCATCCTGCACCACCAACT‐3′ and 5′‐AACACGGAAGGCCATGCCAG‐3′. The temperature profile consisted of initial denaturation at 95 °C for 60 s, followed by 40 cycles of denaturation at 95 °C for 5 s, annealing and elongation at 62 °C for 30 s, and fluorescence monitoring at 60 °C. The specificity of the amplification reaction was determined by performing a standard curve analysis and a melting curve analysis. Relative signal quantification was achieved by normalizing the signal of each gene to that of the Gapdh gene.

### Western blot analysis

Cells were washed with cold PBS, were dissociated by PBS containing 2 mm EDTA, and were centrifuged at 15 000 ***g*** for 30 min. The supernatants were re‐suspended in RIPA lysis buffer including 50 mm Tris/HCl, 150 mm NaCl, 1 mm EDTA, 1 mm EGTA, 1% NP‐40, 10% Glycerol, 20 mm Na_4_P_2_O_7_ 10H_2_O, 200 mm NaF, and 1 mm Na_3_VO_4_. Lysates containing 30 μg proteins were mixed with Lane Marker Sample Buffer (Thermo, Tokyo, Japan) adding 2% beta‐mercaptoethanol for SDS/PAGE. Gels were transferred to Hybond P (GE Healthcare Life Sciences, Tokyo, Japan), and the membranes were blocked in Blocking One (Nacalai) or 5% skimmed milk (Nacalai) in Tris‐buffered saline (TBS) with 0.1% Tween 20 for 1 h and incubated overnight at 4 °C with the antibodies against phospho‐PKC (pan; γ Thr514; Cell Signaling, Tokyo, Japan), phospho‐Hsf1 (Ser303; Abcam, Tokyo, Japan), Hsp70 (Abcam), phospho‐Akt (Ser473; Cell Signaling), Akt (Cell Signaling), phospho‐mTOR (Ser2448; Cell Signaling), mTOR (Cell Signaling), phospho‐S6K1 (Thr389; Cell Signaling), S6K1 (Cell Signaling), phospho‐Eif4ebp1 (Thr37/46; Cell Signaling), Eif4ebp1 (Cell Signaling), phospho‐Foxo3 (Ser253; Cell Signaling), Foxo3 (Cell Signaling), Mef2d (Abcam), Myf5 (Abcam), Myf6 (Santa Cruz, Dallas, TX, USA), Myod1 (Abcam) at a 1 : 1000 dilution, beta‐Actin (Novus Biologicals, Littleton, CO, USA) at a 1 : 200 000 dilution, and Gapdh (Novus Biologicals) at a 1 : 500 000 dilution. The membranes were then washed with TBS with 0.1% Tween 20 for 30 min and incubated with goat anti‐mouse, goat anti‐rat, donkey anti‐rabbit, or donkey anti‐goat IgG horseradish peroxidase‐conjugated antibody (Santa Cruz) at a 1 : 5000 dilution, and the blots were developed with a Chemi‐Lumi One Super (Nacalai) and visualized by ChemiDoxXRS‐J (Bio‐Rad, Hercules, CA, USA) and Lumino Graph I (ATTO, Tokyo, Japan). The intensity of each developed band was measured using a software Quantity One (Bio‐Rad) and CS Analyzer 4 (ATTO). Quantitative analysis was achieved by normalizing the signal of each protein to that of Gapdh or beta‐Actin as previously described 51.

### Statistical analysis

All results are expressed as means ± SD. Data were evaluated for statistical significance by an ANOVA and a Bonferonni adjustment applied to the results of a *t*‐test using spss statistics software, IBM, Tokyo, Japan. *P* values of < 0.05 were regarded as statistically significant.

## Author contributions

Experiments were performed at the Dokkyo Medical University. SO and TN were responsible for the study design. SO was involved in the acquisition and analysis of the data. All authors contributed to the interpretation of the data. SO, TN, and TI drafted the article. All authors agreed to be accountable for all aspects of the work. All persons designated as authors qualify for authorship.

## Conflict of interest

The authors declare no conflict of interest.
